# Segmental vitiligo distribution follows the underlying arterial blood supply territory: a hypothesis based on anatomo-clinical, pathological and physio-pathological studies

**DOI:** 10.3389/fmed.2024.1424887

**Published:** 2024-09-18

**Authors:** Laila Benzekri, Muriel Cario-André, Fatima Zahrae Laamrani, Yvon Gauthier

**Affiliations:** ^1^Dermatology Department, Pigmentary Disorders Outpatient Clinic, Ibn Sina Teaching Hospital, Mohammed V University in Rabat, Rabat, Morocco; ^2^Bordeaux University, INSERM, BRIC, Bordeaux, France; ^3^National Reference Center for Rare Skin Diseases, Bordeaux University Hospital, Bordeaux, France; ^4^Radiology Department, Ibn Sina Teaching Hospital, Mohammed V University in Rabat, Rabat, Morocco; ^5^Vitiligo and Melasma Research Association, Bordeaux, France

**Keywords:** segmental vitiligo, anatomical correspondence, arterial blood-supply, thermography, depigmentation, varicella-zoster virus, periarterial sympathetic nervous system

## Abstract

**Background:**

Segmental vitiligo (SV) is a subset of vitiligo typically characterized by its unilateral distribution. The pathogenesis of SV remains unclear, and until now the two main patterns proposed for SV have lacked biological support. This calls for a new approach.

**Objectives:**

Use data obtained from anatomo-clinical, pathological, and physio-pathological studies to formulate a new hypothesis on segmental vitiligo distribution and its pathogenesis.

**Methods:**

Using transparent templates of local arterial blood supply, we evaluated anatomical correspondence (AC) in 140 SV lesions according to the number of SV lesions that fit within the corresponding arterial blood-supply areas. SV lesions were graded as 1 (moderate: AC < 50%), 2 (good: AC > 50%), or 3 (excellent: AC of all lesions). To support this anatomical investigation, we searched for complementary assessments according to the activity of SV lesions. Arterial and periarterial network impairment and inflammatory infiltration were histologically studied using nerve growth factor (NGF) and CD4 and CD8 monoclonal antibodies. Increased blood flow of the underlying arteries was also investigated using thermography and ultrasonography.

**Results:**

We recruited 140 patients with a sex ratio of 0.8 and mean age 26.13 years. Localizations: head and neck 84.28%; trunk 6.42%; upper limb 5%; genital areas 2.14%; lower limb 1.42%. The AC of each SV lesion with the underlying artery blood supply territory was rated as 72% excellent; 16% good; and 12% moderate. Histologically (40 patients), we found some periarterial network impairments. Thermal asymmetry was significantly associated with active SV (*p* < 0.001).

**Conclusion:**

We hypothesized that SV distribution corresponds to the underlying artery blood-supply territory.

## 1 Introduction

Segmental vitiligo (SV) is a subset of vitiligo primarily characterized by a unilateral distribution (1). After rapid progression, SV tends to stabilize within the first 2 years of onset. However, some patients experience disease recurrence despite treatment (2). The pathogenesis of SV remains unclear. Clinically, the various sites affected by depigmentation recur according to characteristic patterns (3–12). Increasing our knowledge of SV distribution patterns may be crucial for understanding the pathogenesis of SV and thereby predicting potential sites of depigmentation extension.

**Figure 1:**
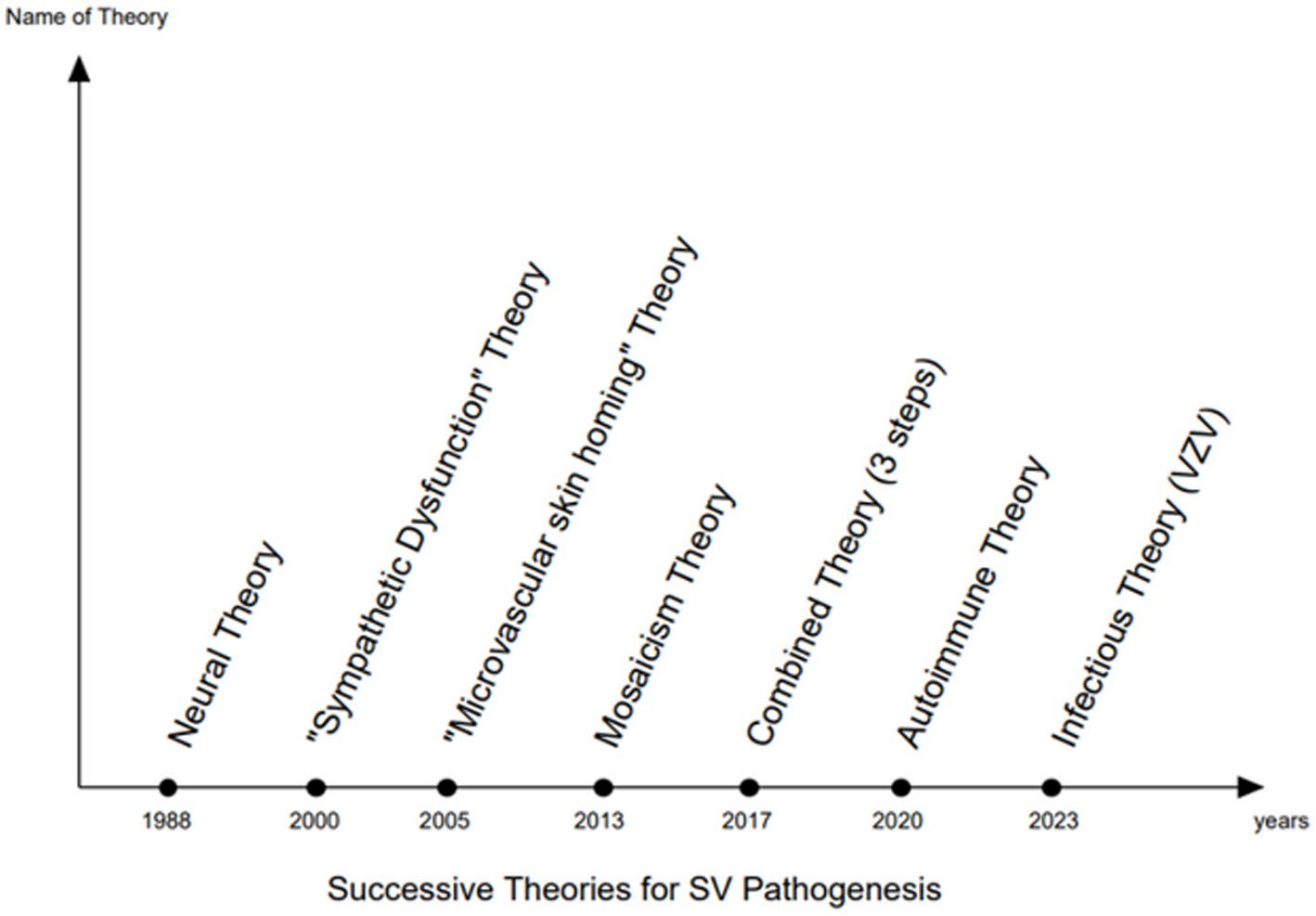
SV pathogenesis: successive theories.

Six theories regarding the pathogenesis of SV have been proposed to date (Figure 1):

- A neuronal theory focused on the dermatomal distribution (13)
- A sympathetic dysfunction theory (14, 15)
- A microvascular skin homing theory based on a hypothetical migration pattern of cytotoxic T cells along the microvascular system (16)
- A somatic mosaicism theory based on similarities between the SV lesion orientation/distribution and certain mosaic skin disorders (17, 18)
- A convergent theory involving successive aetiopathologic pathways (e.g., neuronal pathways, mosaicism, and microvascular skin homing) (19)
- An autoimmune theory based on CD8-T lymphocyte infiltration and the possible onset of mixed vitiligo (20)
- An infectious theory involving anatomically corresponding sympathetic fibers and melanocytes affected by the varicella-zoster virus (VZV) (21, 22)

The variation among these theories indicates the need for a new clinical, histological, and physio-pathological approach.

A clinical study of the profile of patients with SV demonstrated that many SV lesions with a ‘quasi-dermatomal pattern’ did not precisely fit within the dermatomal areas, nor were any sensory dysfunctions found in these areas. Moreover, numerous typical or atypical patterns on the face and trunk did not precisely follow Blaschko’s lines, as in mosaic skin disorders. Thus, with these two clinical theories based exclusively on lesion morphology and orientation, 29 to 40% of SV lesions remain unclassified (23). Several authors have noted the lower incidence of a family history and coexisting autoimmune disorders, highlighting the long-term efficacy of dermato-surgery compared with non-SV. This suggests that autoimmunity may not play a significant role in the pathogenesis of SV.

Histologically, besides melanocyte loss, a variable inflammatory infiltrate with CD8-T lymphocytes located in the epidermis and superficial dermis, as well as some viral cytopathic effects in the epidermis, were temporarily found with routine histology. Additionally, during the extensive period, a positive immunoreactivity against VZV was found in the epidermis. Some characteristic VZV virions were detected by electron microscopy in melanocyte cytoplasm and periarterial nerve fibers (21, 22). Typically, inflammatory infiltrates and virions are not observed in stable SV lesions. The inflammatory infiltrate with CD8-T lymphocytes temporarily found in the epidermis and superficial dermis could be an inflammatory reaction against local viral infestation. The incidence of mixed vitiligo (SV associated with autoimmune non-SV) is low and may result from accidental immunization against melanocytes previously killed by the virus.

Physio-pathologically, it is important to note that molecular analysis data regarding the mosaicism theory (24), the microvascular skin homing theory, and the convergent theory are still missing. Besides melanocyte loss, the main abnormality described in the lesional skin of patients with SV affects periarterial sympathetic function. This includes altered acetylcholine activity, increased catecholamine and neuropeptide release (25–27), and increased cutaneous blood flow compared with the contralateral normal skin, along with a significantly increased α-and β-adrenoreceptor response in SV lesions (14). Moreover, a virus reactivated in the anatomically corresponding sympathetic ganglia could reach the periarterial sympathetic network. During this process, the virus could trigger dysfunction in both arterial vaso-motricity and the cell machinery of infected melanocytes, inducing local depigmentation.

Until now, the possibility of an SV distribution following the cutaneous arterial blood supply has never been considered. Therefore, the purposes of our study were to investigate any anatomical correspondence between the pattern distribution of SV and arterial vessel mapping, detect any functional impairments of the local underlying arteries, and propose a new approach to the pathogenesis of SV.

### 1.1 Contribution statement

Segmental vitiligo (SV) is a subset of vitiligo that in its main form is characterized by a unilateral distribution. The SV pathogenesis remains unclear. To date, the two main distribution patterns proposed for SV (according to « Neural », or « Mosaicism » theories) appeared to be in conflict and in any cases unconvincing for several reasons. 1 The lesions distribution did not fit precisely dermatoma areas and no sensory dysfunction was found in these areas. 2 These two theories which were presented without any biological support were purely speculative. This calls for a new approach. Previous studies reported a cutaneous blood flow and adrenoreceptor response in SV. Consequently, we hypothesized that the underlying cutaneous arterial vessels could be implicated in the development of SV lesions. At the end of the study, a significative anatomical correspondence (AC) between the SV distribution and the underlying arterial mapping was found in 72% of cases and was associated with an arterial dysfunction in 62.07% of cases. These data lead us to formulate a new hypothesis on SV distribution and on its pathogenesis. Clinically, the extension territory of SV lesions could be predicted. Physio-pathologically, the knowledge and the correction of the causes of the arterial dysfunctions could improve in the future the SV management.

## 2 Methods

### 2.1 Patients and methods

Patients who had SV at different sites of the whole body were recruited at the Pigmentary Disorders Outpatient Clinic of the Department of Dermatology at Ibn Sina Hospital in Rabat, Morocco between January 1, 2018 and December 31, 2022. The study was approved by the local ethics committee of Mohammed V University, in Rabat, Morocco. All included patients provided written informed consent for the use of their medical data. Data were based on clinical examination of the whole body in addition to Wood’s lamp examination and photo-analysis of all lesions.

### 2.2 Clinical and anatomical study

Digital pictures of local arterial blood supply areas and of SV lesions were taken. Arterial blood supply areas were traced with red ink on transparent paper directly from the digital pictures of anatomical maps (28) (Supplementary Figure S1). The choice of arterial blood supply map depended on the location of SV. In the same manner, the borders of SV lesions were underlined with black ink directly from the digital pictures. For standardizing the size of local arterial blood supply and segmental vitiligo areas, the help of a zoom in and out function was used. After the superimposition of the two transparent papers, we evaluated anatomical correspondence (AC) in 140 SV lesions according to the number of SV lesions that fit within the corresponding arterial blood-supply areas. SV lesions were graded as 1 (moderate: AC < 50%), 2 (good: AC > 50%), or 3 (excellent: AC of all lesions). The assessment was performed by two investigators (L. Benzekri and Y. Gauthier). Lesions were re-evaluated if there was disagreement between the two investigators.

### 2.3 Histological study

Histological, immunochemical studies of skin samples from the margin were performed using HES staining to detect dermal infiltrate and analyze dermal arteriole features. The perivascular network was studied using Nerve Growth Factor (NGF).

### 2.4 Physiopathological study

#### 2.4.1 Thermography

Blood flow and heat patterns were assessed through various areas using a Trotec R infrared camera with a 160 ×120 pixel detector and a spectral range of 8–14 μm. Thermography was performed by a single examiner in a room with no direct sunlight or incandescent light. The temperature of the environment was approximatively 20°C.

Thus, in 29 patients who were investigated with thermography, a semi-quantitative analysis focusing on whether or not thermal asymmetry occurred was possible.

#### 2.4.2 Doppler ultrasonography

Ultrasound Doppler flowmetry was used to evaluate the cutaneous blood flow over SV lesions and contralateral (normal) skin (29, 30). The average values for resistive index (RI) of the anatomically corresponding arteries with SV was performed. A decrease in the RI usually reflects increased flow.

### 2.5 Statistical analysis

Data are presented as the mean ± standard deviation (SD) for variables with a normal distribution, and as the median and interquartile range for variables with skewed distributions. Statistical differences between groups were evaluated by the chi-square test or Fisher’s exact test for categorical variables. A two-tailed *p* value <0.05 was considered significant. Statistical analyses were carried out using using Jamovi software version 2.3.16.

## 3 Results

### 3.1 Study population

In total, 140 patients were recruited for analysis in the Pigmentary Disorders Outpatient Clinic of the Department of Dermatology (Ibn Sina Hospital, Rabat, Morocco). The sex ratio was 0.8, and the age range of patients was 3–73, with a mean age of 26.13 years ±14.88. At the first visit, the median age of SV lesions was 12 months. Disease activity was considered positive if new lesions had been detected in the past 4 months.

### 3.2 Anatomical analysis

**Figure 2:**
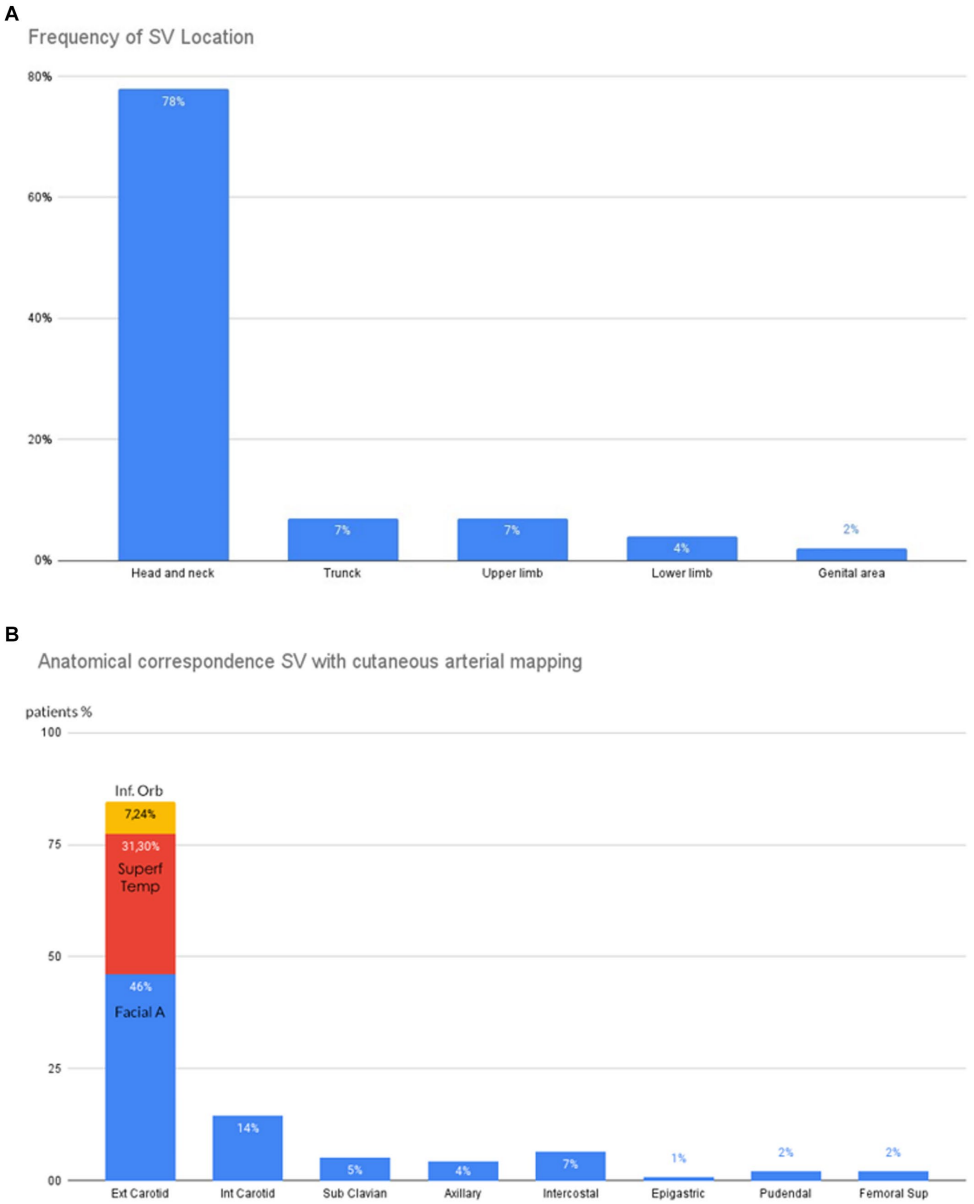
Clinical characteristics of SV Patients. **(A)** Distribution of SV. **(B)** Anatomical correspondences between SV and underlying arteries. Ext.Carotid, External Carotid; Inter.Carotid, Internal Carotid.

The head and neck were the initial sites of onset in 84.28% of patients. The trunk and upper limb were the sites for 6.42 and 5% of cases, respectively, followed by the genital areas in 2.14% of cases and the lower limb in 1.42% (Figures 2A,B). The AC of each SV lesion with the underlying artery blood-supply territory was excellent in 72% of cases, good in 16% of cases, and moderate in 12% of cases.

**Figure 3:**
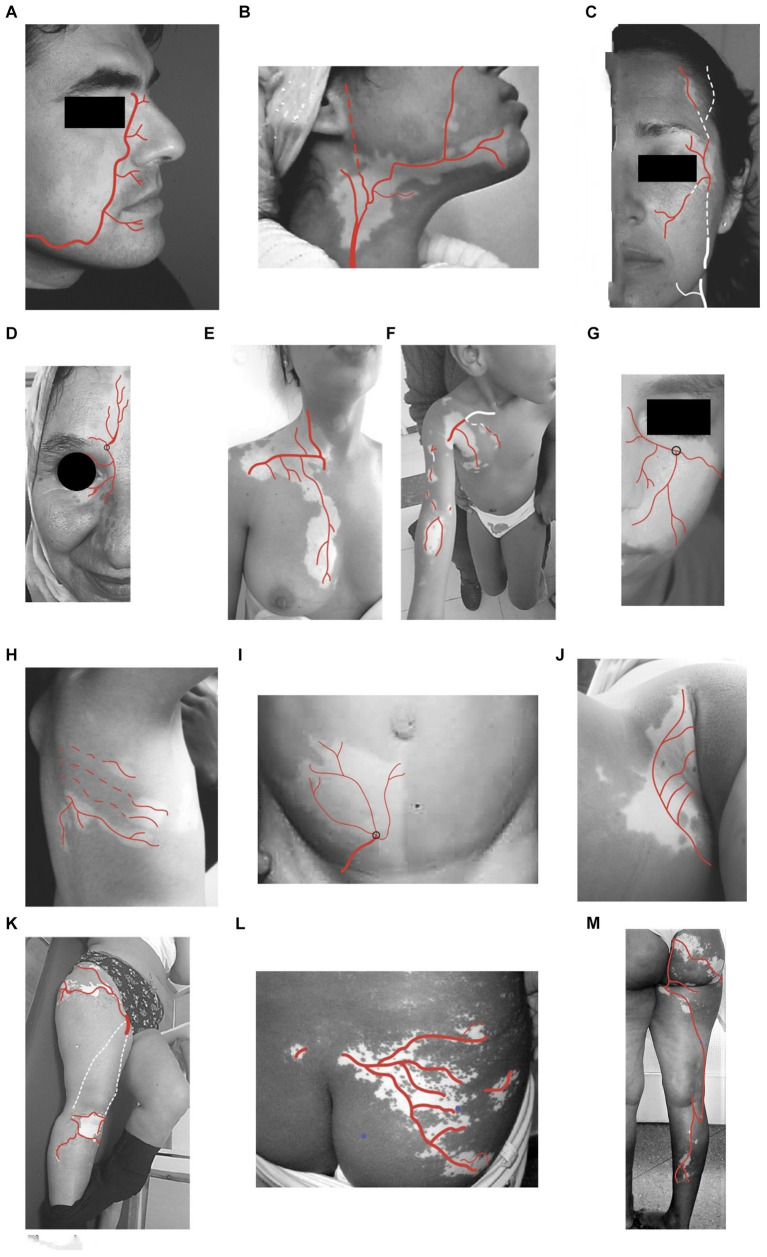
Anatomical correspondences (AC) of SV pattern and cutaneous underlying arteries. **(A)** With distal *facial artery* **(B)** with proximal *facial and sub-mental arteries* **(C)** with *superficial temporal artery.* **(D)** with *supraorbital artery* **(E)** with *internal thoracic artery (internal mammary artery), external thoracic artery* **(F)** with *superficial branches of humeral, radial, and ulnar arteries* **(G)** with *infraorbital artery* **(H)** with *branches of intercostal arteries* **(I)** with *superficial branches of epigastric artery* **(J)** with *superficial pudendal artery* **(K)** with *superficial circumflex arteries and genicular arteries.* **(L)** with branches of *superficial gluteal arteries* **(M)** with *superficial gluteal arteries, perforating branches of profunda femoris, posterior tibial arteries.*

The SV lesions correspond anatomically to either the course of the entire trunk of the underlying nutrient artery and its branches or to the partial course of a distal segment or one or a few branches of this nutrient artery. The depigmentation is observed according either to a *single pattern* (133 cases) corresponding to a single main artery (Figure 3) or a *combined pattern* due to several anastomoses between some main arteries and their branches (Supplementary Figure S2).

*In the head and neck, AC correspondence is found with:*

1. The facial artery course (Figure 3A). This is the most frequent subtype (*n* = 67 cases, 50.37%);
2. The proximal facial artery course (Figure 3B; *n =* 4 cases, 3%);
3. The superficial temporal artery course (Figure 3C; *n* = 19 cases, 14.28%);
4. The supraorbital artery course (Figure 3D; *n* = 5 cases, 3.75%);
5. The infra-orbital artery course (Figure 3G; *n* = 16 cases, 12.03%).

*Seven combined patterns occur in the face due to the frequent anastomoses between.*

*The secondary branches of the EC and IC.*

*In the thorax, AC is found with:*

1. The subclavian artery course with its branches (Figure 3E; *n* = 4 cases, 3%);
2. The posterior and anterior intercostal artery course branches of the internal thoracic (Figure 3I; *n* = 5 cases, 3.75%).

*In the upper limb, AC is found with:*

The axillary artery course and its branches (Figure 3F; *n* = 7 cases, 5.26%).

*In the lower part of the abdomen, AC is found with:*

The epigastric arteries and deep circumflex arteries (branches of external iliac Artery; Figure 3J; *n* = 1 case, 0.75%).

*In the buttocks and genital areas, AC is found with:*

1. The distal branches of the superior and inferior gluteal arteries (Figure 3M; *n* = 1 case, 0.75%);
2. The internal pudendal artery (Figure 3K; *n* = 2 cases, 1.5%).

*In the lower limb, AC is found with:*

1. The circumflex arteries and the superficial femoral artery (Figure 3L; *n* = 1 case, 0.75%);
2. The superficial gluteal arteries (Figure 3M; *n* = 1 case, 0.75%).

### 3.3 Histological study (40 patients)

**Figure 4:**
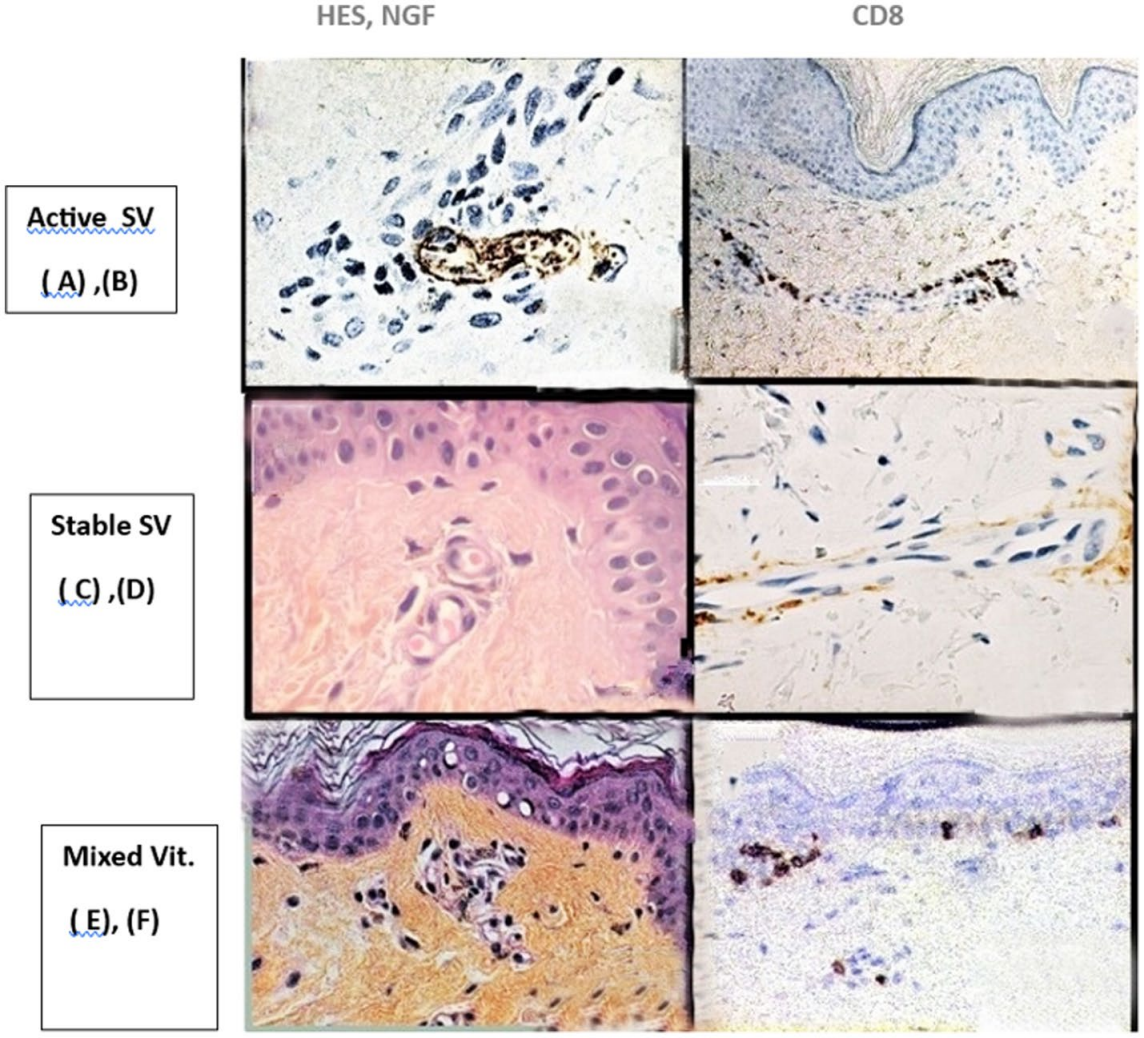
Histological findings according to the age of the SV lesions. Active SV **(A)** Perinervous lymphocytic infiltrate in the dermis. Staining NGF, X40 magnification, **(B)** CD8 Perivascular infiltrate. Immunolabeling with CD8-Lymphocyte mono clonal antibody, x20 magnification. Stable SV **(C)** passive vasodilatation of vessels, without inflammatory infiltrate. HES staining, x 40 magnification, **(D)** Absence of perivascular inflammatory infiltrate, CD8 immunostaining,x20 magnification. Mixed vitiligo **(E)** Peri-vascular lymhocytic infiltrate with melanophages. HES staining; x40 magnification, **(F)** CD8 infiltrate of superficial dermis and epidermis. CD8 immunolabeling, x20 magnification. SV, segmental vitiligo; HES, Hematein Eosin Safran; NGF, Nerve Growth Factor.

Dynamic changes in histological findings according to the age of SV lesions were noted (Figure 4).

*In recent and active SV:* A moderate perineural and perivascular inflammatory infiltrate, including CD8 lymphocytes, was found in 13 cases (32.5%) (Figures 4A,B).

*In long-lasting and stable SV:* Inflammatory infiltrate in the dermis was discrete or absent. Also noted were dilatation of superficial vessels and degenerative alterations in cutaneous nerves with a swelling and disruption of the perivascular network in 24 cases (60%) (Figures 4C,D).

*In mixed vitiligo* (segmental associated with non-segmental vitiligo; *n* = 3, 7.5%): A mild superficial lymphocytic infiltrate that included CD4 and CD8 lymphocytes and melanophages was frequently observed just below a lichenoid infiltrate around melanocytes in the epidermis (Figures 4E,F).

### 3.4 Physio-pathological studies

**Figure 5:**
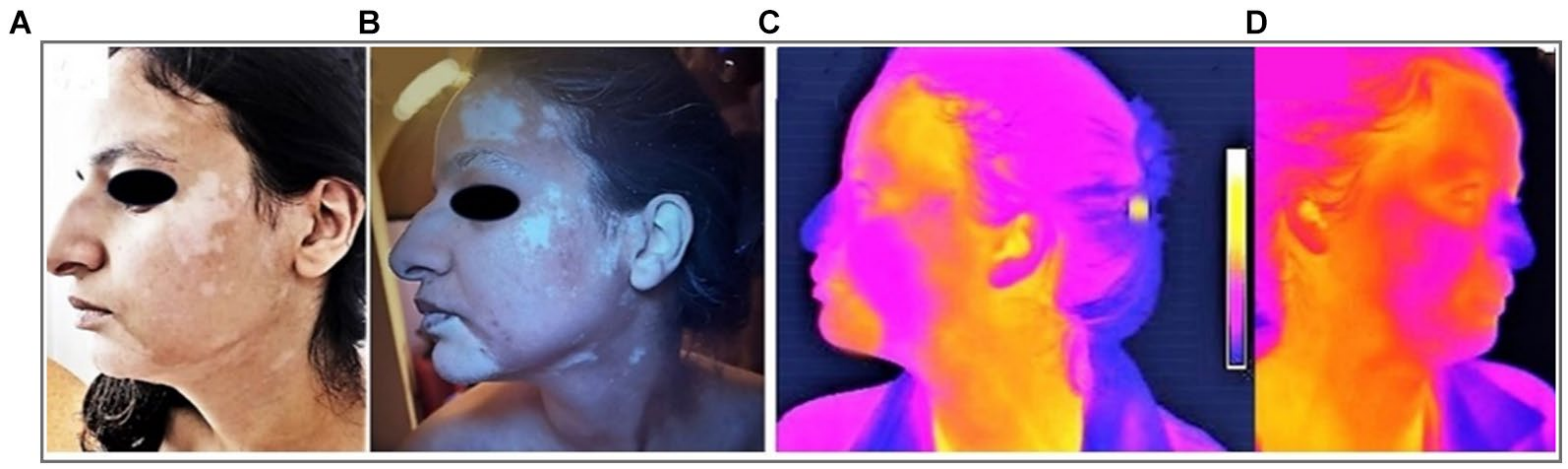
Thermal asymmetry in facial SV assessed with thermography. **(A)** Combined SV of the face located in the left side **(B)** Wood’s lamp examination of the left side **(C)** With thermography, warmer areas in forehead, chin, upper part of the cheek in the left side **(D)** Normal aspect of the thermography in the right side.

The SV lesions showed different blood flow profiles, suggesting some arterial dysfunction when compared with contralateral normal skin. Twenty nine patients underwent thermography. The entire evolution of SV is marked by dynamic changes in skin temperature. In 18 cases, (62.07%), thermal asymmetry was found (Figure 5) with a median temperature increase in SV lesion of 1.4° [1°; 1.5°]. Nineteen cases (65.52%) of SV were active. Thermal asymmetry was significantly associated with active SV (*p* < 0.001).

With ultrasonography (22 patients), a decrease in RI, which reflects an increase in blood flow in corresponding arteries, was found in only eight cases (35%). An equivalent RI from both the SV area and contralateral side was found in 14 cases (65%).

## 4 Discussion

The main goal of the present study was to shed insight on the existence of a possible link between the underlying cutaneous arterial mapping and the SV clinical distribution. Moreover, these data can lead to the proposal of a new approach to SV pathogenesis.

### 4.1 Anatomical correspondence

Based on the anatomical analysis,” *a*t*ypical patterns”* can be due to the existence of numerous anastomoses between adjacent arteries, for example:

1. In bilateral SV as well as probably when SV crosses the midline, there are anastomoses between the right and left homonymous arteries;
2. Hemifacial SV can depend on the anastomoses existing between several branches of external and internal carotids;
3. Checkerboard, hemicorporeal, and phylloid SV can correspond to the anastomoses between homolateral and multilayered arteries.

In other parts, a *“quasi-dermatomal pattern*” can be designated when periarterial autonomic nerves travel together with sensory nerves (i.e., through cranial foramens and in intercostal spaces), and a *“Blaschkoid pattern*” can be designated only when arteries follow the same oblique direction as that of Blaschko lines (i.e., in the facial, superficial temporal, and intercostal arteries).

**Figure 6:**
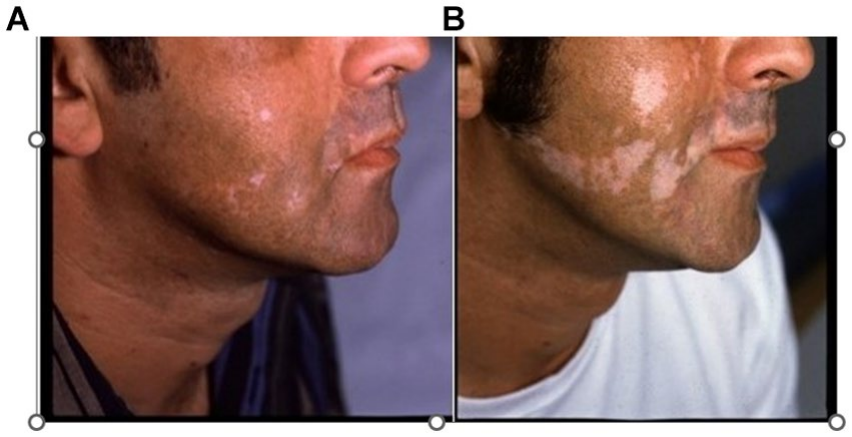
Extension of SV lesions following the course of underlying arteries **(A)** Depigmentation mainly involving the superior lip and the cheek **(B)** Extension of the depigmentation following exactly the course of the facial artery and its branch the superior labial artery.

### 4.2 Implication of underlying arterial vessels

The implication of arterial vessels in SV was previously hypothesized in the “micro-vascular skin homing” theory. This theory suggested that the midline delineation of a unilateral lesion could represent the migration pattern of cytotoxic T-cells from anatomically corresponding lymph nodes along the microvascular system (16). Unfortunately, this hypothesis was purely speculative and lacked any histological support. Thus, in our study, another process of artery involvement in SV is provided by the simultaneous histopathological and physio-pathological investigations. The histological study successively shows (Figure 4):

1. A periarterial and perineural location of the lymphocytic infiltrate observed in active and very recent SV.
2. A passive vasodilatation of superficial arterioles and a degenerative alteration of periarterial nerves that was noted in stable SV.

These data are in complete agreement with a previous study that demonstrated in SV lesions an increase in adrenoreceptors and blood flow.

### 4.3 Arterial dysfunction causes

To understand this local arterial dysfunction, several biochemical investigations related to neural mediators were previously performed in SV, but they were contradictory and must be considered unconclusive (29, 30). Thus, a viral implication was recently proposed for SV pathogenesis for many reasons:

1. In several cases, SV was associated with viral encephalitis and transverse myelitis (31–33).
2. VZV was detected with PCR in a few cases of unilateral choroidal vitiligo which appears following the arterial vessels, as in cutaneous lesions (34–36).
3. In an ultrastructural study, we found in three cases characteristic VZV virions within damaged melanocytes, which were staying at the margins of recent SV lesions (21, 22).
4. *In vitro*, a tropism of VZV for melanocyte was suggested. VZV hijacks the melanogenic system of cultured pigment cells inducing a probably lethal dysfunction (37).

### 4.4 A new approach of SV pathogenesis

**Figure 7:**
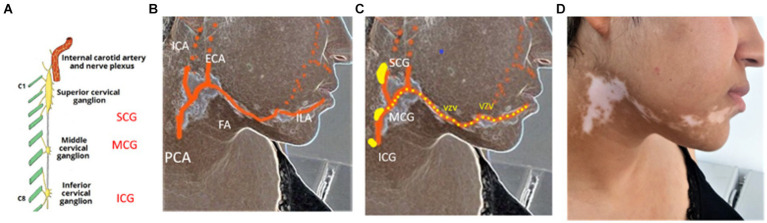
Possible pathogenesis of a SV developed in external carotid artery blood supply area **(A)** Sympathetic cervical ganglia-Superior cervical ganglion (SCG)-Middle cervical ganglion (MCG)-Inferior cervical ganglion (ICG) **(B)** Blood supply area of External carotid artery (ECA) and its branches: facial (FA) and inferior labial (ILA) arteries. PCA, primitive carotid artery; ICA, internal carotid artery. **(C)** Possible migration route (yellow dots) of varicella-zoster viruses (VZV) reactivated in MCG through periarterial sympathetic network along the course of ECA and its branches **(D)** DSV, depigmented sequelae secondary to the brief VZV migration along the ECA and its branches.

Finally, we can hypothesize that each SV pattern distribution could reveal the peripheral vascular routes followed by reactivated viruses through periarterial sympathetic nerves from sympathetic ganglia toward the epidermal melanocyte (Figures 6,7). SV could be considered as depigmented sequelae secondary to a silentious low rate and short duration of viral infection traveling along the periarterial sympathetic nervous system. Exceptionally, some damaged melanocytes that drop down into the dermis could trigger an autoimmune attack and the destruction of the epidermal melanocytes by CD8 lymphocytes, as described in mixed vitiligo (38, 39).

This study has some limitations that should be noted. It is a cross-sectional single-center observational study. Moreover, it was not possible for us to perform laser Doppler flowmetry or iontophoresis, which are more accurate for cutaneous microcirculatory assessment than ultrasonography.

## 5 Conclusion

For the first time, an evident AC is shown between depigmentation distribution and underlying arterial vessel course, which could shed light on SV pathogenesis.

## Data Availability

The datasets presented in this article are not readily available because the original contributions presented in the study are included in the article/Supplementary materials, further inquiries can be directed to the corresponding author. Requests to access the datasets should be directed to benzekrilaila@yahoo.fr.
