# Supplementary material for: Segmental vitiligo distribution follows the underlying arterial blood supply territory: a hypothesis based on anatomo-clinical, pathological and physio-pathological studies
Source: Front Med (Lausanne). 2024 Sep 18;11:1424887. doi: 10.3389/fmed.2024.1424887 (PMC11445008; doi:10.3389/fmed.2024.1424887)
Supplement: Supplementary file 1 [file Data_Sheet_1.docx]

**Supplementary file :**

**
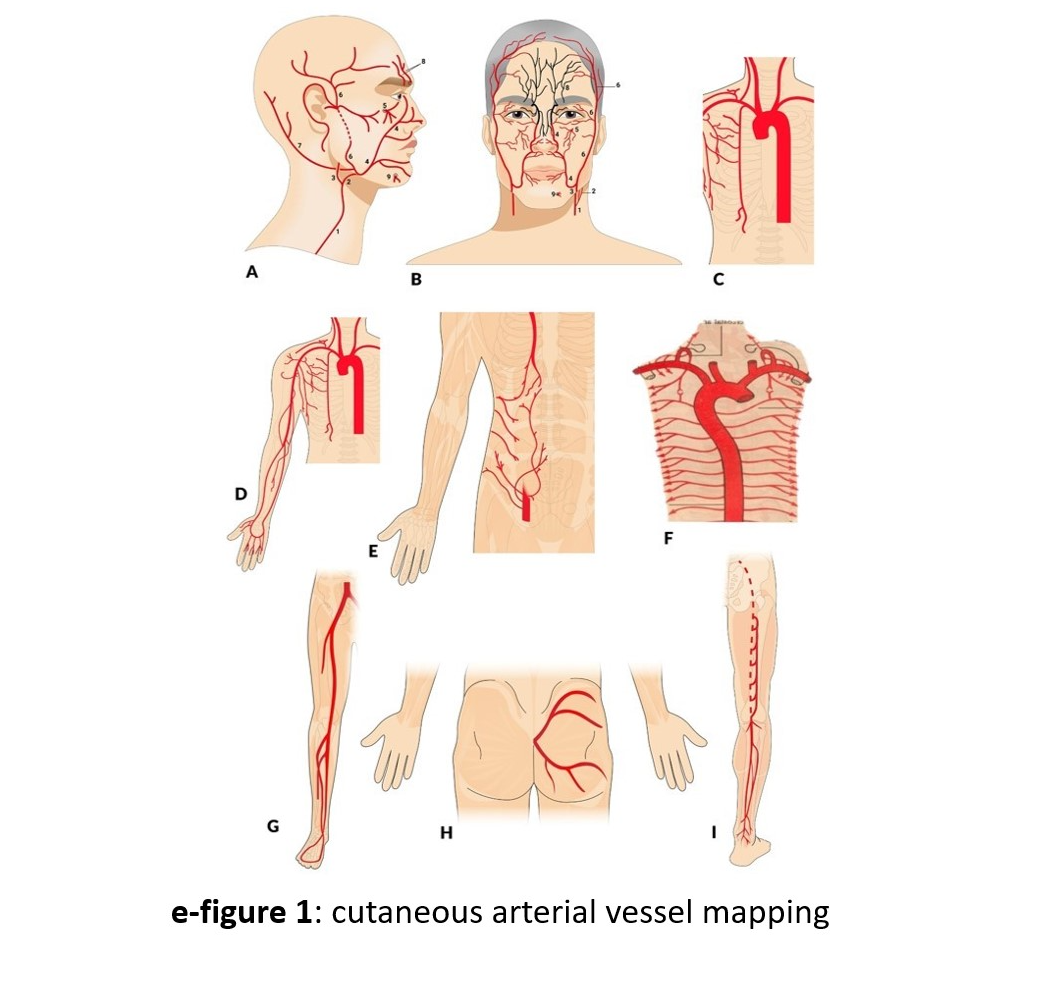
**

***SM 1: Cutaneous arterial mapping***

A/ Lateral side of the face is supplied by the branches of: common carotid, external carotid, internal carotid, facial, submental, inferior labial, superior labial, superficial temporal, infraorbital, supraorbital arteries

B/ In addition to the external carotid branches, internal carotid provides blood supply through ophtalmic artery to the skin of anterior part of the face (fore head, eyelid and lateral side of the nose) through supraorbital and supratrochlear arteries

C/ Anterior thorax wall is supplied by the branches of subclavian artery a mainly the internal thoracic (internal mammary), the external thoracic arteries.

D / Posterior thoracic wall is supplied by the superficial branches arising from thoracic aort, subclavian, axillar and intercostal arteries

D/ Upper limb is supplied by the superficial branches of subclavian, axillary, humeral, radial and ulnear arteries.

E/ Lower wall of abdomen is supplied by the superficial superior and inferior epigastric arteries, sub costal and superficial circonflex arteries.

F/ Anterior lower limb is supplied by the superficial branches of epigastric, pudendal, circumflex, femoral arteries for the tigh, genicular arteries for the knee and anterior tibial artery for the leg.

G/ Posterior lower limb is supplied by: branches of superior and inferior gluteal arteries for the gluteal region, perforating branches of the profunda femoris artery for the thigh region, popliteal, posterior tibial, fibular arteries for the leg

**
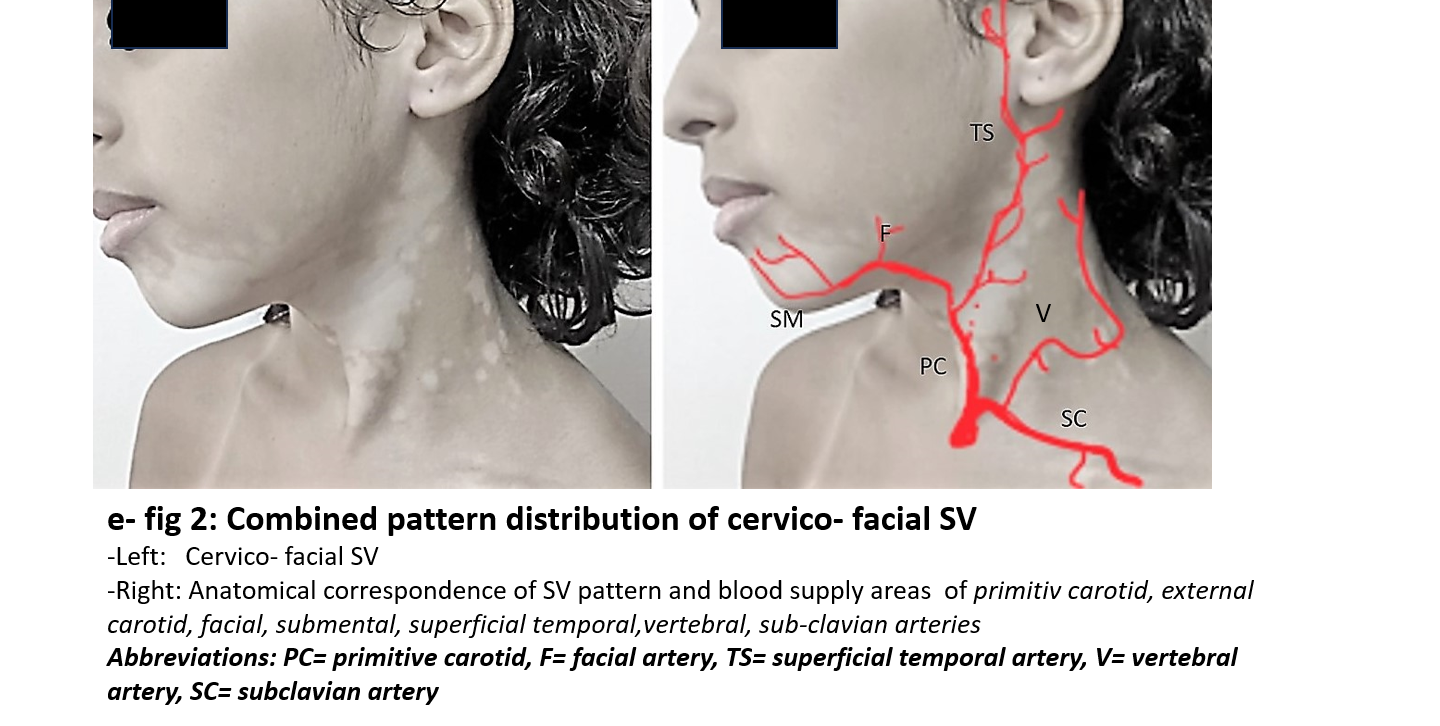
**

**SM2: Combined pattern**
